# Remifentanil target-controlled infusion with intranasal dexmedetomidine for vitreoretinal procedures: a randomized controlled trial

**DOI:** 10.3325/cmj.2021.62.233

**Published:** 2021-06

**Authors:** Iztok Potočnik, Lea Andjelkovič-Juvan, Andrej Hostnik, Jasmina Markovič-Božič

**Affiliations:** 1 Department of Anesthesiology and Intensive Care, Institute of Oncology Ljubljana, Ljubljana, Slovenia; 2 Medical Faculty, University of Ljubljana, Ljubljana, Slovenia; 3 Clinical Department of Anesthesiology and Intensive Therapy, University Medical Center Ljubljana, Ljubljana, Slovenia

## Abstract

**Aim:**

To evaluate the consumption of remifentanil (as a primary end-point), analgesia, sedation, hemodynamics, respiratory effects, and surgeon and patient satisfaction (as a secondary end-point) with dexmedetomidine sedation compared with those of remifentanil sedation in patients undergoing vitreoretinal surgery.

**Methods:**

Patients subjected to retinal ophthalmic surgical procedures were randomized to one of two intraoperative sedation groups: one group (n = 21) received intranasal dexmedetomidine plus intravenous remifentanil (DEX-REMI group), and the other group (n = 19) received intravenous remifentanil only (REM group). The treatment was placebo-controlled. The sedation level was controlled according to the bispectral index, with target values between 80%-90%. Patient levels of comfort, sedation, and pain were documented. The number of intraoperative complications and the level of satisfaction were assessed. Remifentanil consumption and hemodynamic parameters were also included in the statistical analysis.

**Results:**

The level of remifentanil consumption was significantly lower in the DEX-REMI group, but combination sedation improved the surgeon's, anesthesiologist's, and patients' satisfaction scores. Importantly, the number of complications was zero in the DEX-REMI group, while eight cases of complications were noted in the REM group. The DEX-REMI group showed lower mean minimal arterial pressure, but it was still in the normotensive range.

**Conclusions:**

For patients undergoing ophthalmic procedures, sedation with a combination of intranasal dexmedetomidine and an intravenous infusion of remifentanil provides lower remifentanil consumption, better satisfaction scores, and a lower complication rate than sedation with a remifentanil infusion alone.

**Clinical trial number:**

NCT 03251222

Conscious sedation is an established anesthetic method of choice in patients undergoing short ophthalmic procedures and has been used successfully for many years. In Slovenia, there is also a published protocol in use for such cases (1).

Intravenous (i.v.) remifentanil is used for analgesic and sedative purposes. It is a short-acting opioid analgesic with partial anxiolytic action that has been thoroughly studied in obstetric analgesia (2).

The fine intra-ocular endoscopic technique is implemented in vitrectomies. Surgical instruments are inserted into the vitreous humor through the sclera by a surgeon. Patient cooperation is crucial during the procedure, meaning the level of sedation should not be too deep. Otherwise, sudden eye movements could potentially result in eye injury (3). For many ophthalmic surgeons, local anesthesia (LA) has become preferred over general anesthesia (GA) because of quicker patient rehabilitation and avoidance of possible complications from GA (4). Several methods of LA have been described for vitreoretinal cases, including retrobulbar, peribulbar, sub-Tenon’s, and even topical anesthesia (5). Many drugs have been used for sedation during eye surgery, such as propofol, benzodiazepines, and opioids, and there is a relative risk of oversedation, disorientation, and confusion, in addition to an increased risk of respiratory depression and oxygen desaturation (5,6). All of these adverse effects can hamper patient cooperation during surgery and make these agents less than ideal for intraoperative management of sedation. As a result, sedatives and anxiolytics with unpredictable dose requirements, such as propofol and midazolam, are not optimal for such procedures.

In contrast, dexmedetomidine is a highly selective alpha-2-adrenoreceptor agonist with both sedative and analgesic properties and is not associated with respiratory depressant effects. Dexmedetomidine has been studied for its sedation- and analgesia-sparing properties in intensive care and surgical settings (eg, neurosurgery, maxillofacial surgery, ENT surgery) but not in vitreoretinal surgery (7-9).

Dexmedetomidine is mainly administered intravenously. Although intranasal (i.n.) dexmedetomidine is still used off-label, recently an increasing consensus has emerged for its different uses, namely, in non-painful diagnostic procedures, in painful procedures, and in surgical premedication. Some studies have been published regarding this form of use in the pediatric population (10-12). However, at present, there is no consensus regarding indications, dosage, and timing for administration. Available pediatric evidence confirms the efficacy and safety of dexmedetomidine for i.n. administration. The reported dose for pediatric procedures performed under sedation ranges from 2.5 to 4 μg/kg i.n. Onset of action is expected to be slower (25-30-minute) with low doses (1-2 μg/kg) and faster (16.7-28-minute) with higher doses (2.5-3 μg/kg), while offset time is similar in both – 85-minute in average; range 55-100 minutes (13).

The aim of our study was to evaluate the consumption of remifentanil (as a primary end-point), analgesia, sedation, hemodynamics, respiratory effects, and surgeon's and patients' satisfaction (as a secondary end-point) with dexmedetomidine sedation compared with those of remifentanil sedation in patients undergoing vitreoretinal surgery. We conducted an applied research study that produced objective indicators, showing which type of sedation was more comfortable for patients and more effective in terms of achieving the desired sedation and reducing pain during vitreoretinal procedures with fewer side effects.

## METHODS

This randomized, prospective, double-blind study was conducted from Q1 2017-Q3 2018 at the University Medical Center Ljubljana, Department of Anesthesiology and Surgical Intensive Care and Ophthalmology Clinic Ljubljana. The study was approved by the National Medical Ethics Committee of the Republic of Slovenia. All the procedures were performed in accordance with the Declaration of Helsinki. The CONSORT recommendations for reporting randomized trials were followed. All participants gave a written informed consent.

We enrolled 40 cooperative patients aged 18-85 years with American Society of Anesthesiologists (ASA) physical status I-III and Glasgow Coma Scale score of 15 who were scheduled for vitreoretinal procedures. The patients included in the study were operated on by the same surgeon and anaesthetized by the same anesthesiologist.

The included patients were appropriately and thoroughly informed about the trial. They were able to freely withdraw from the trial without consequences, even after deciding to take part in it.

Patients were excluded if they (a) wanted general anesthesia, (b) were unable to lie flat for a prolonged period of time, (c) had severe hearing impairment, claustrophobia, nystagmus, re-operation of the affected eye, or a monocle, (d) had contraindications to receiving dexmedetomidine (drug hypersensitivity, second- or third-degree atrioventricular block, uncontrolled arterial hypertension and cerebrovascular disease), (e) had severe cardiac failure (NYHA>3), pulmonary obstructive disease (FEV_1_<40%), or psychiatric diseases, (f) were taking psychotropic drugs, or (g) had received beta-blockers before the surgery.

Using a computer-generated list, the patients were randomized to one of the two groups by the third author, who was not involved in patient care. The first author enrolled the patients and informed them about study participation. The trial was double-blind, and neither the anesthesiologist nor the surgeon or patients knew whether the patient was given 0.9% i.n. saline or dexmedetomidine.

Anesthesia technique was the same as that used in other patients undergoing these procedures, in accordance with the University Medical Center Ljubljana’s standardized operational protocol (1): standard monitoring (electrocardiography [ECG], pulse oximetry, noninvasive blood pressure measurement every three minutes), bi-nasal catheterization for oxygenation and capnometry during the procedure, i.v. cannulation with an i.v. Hartmann solution, and use of a heating pad. Patients were not premedicated; they were given i.v. dexamethasone 4 mg as an antiemetic.

Patients in the REMI group were given 1 mL of 0.9% i.n. saline 30 minutes before the surgical procedure. They were sedated with i.v. remifentanil administered via a target-controlled infusion pump (Fresenius Kabi Orchestra, Bad Homburg, Germany). The target effect site concentration (Ce) was 1-3 ng mL^−1^. The infusion was started 10 minutes before the surgical procedure. The sedation depth was measured with the bispectral target index (BIS), with the target BIS being 80%-90%.

Patients in the DEX-REMI group were given 1 μg per kg of body mass of i.n. dexmedetomidine 30 minutes before the procedure. After that, they were given i.v. remifentanil, with the same target Ce (1-3 ng mL^−1^) and BIS (80%-90%) values as the REMI group.

In the event of bradycardia (heart rate [HR]<50 minutes^−1^), patients received 0.01 mg kg^−1^ of atropine. If the systolic blood pressure and HR increased by more than 30% from baseline, and the patients did not have pain or discomfort, we planned to use urapidil or metoprolol, as appropriate.

At the end of procedure, the remifentanil infusion was stopped in both groups. Afterwards, patients were relocated to the recovery unit. The primary outcome measure was remifentanil consumption. The secondary outcome measures were sedation, pain, comfort, satisfaction, and complications.

We collected patients' demographics and other data (comorbidities, regular medications used). The cumulative doses of dexmedetomidine, remifentanil, and other drugs given during the procedures were documented.

During the surgery we monitored patients’ blood pressure (non-invasively) and HR. At the end, the maximum and minimum blood pressures and HRs observed during procedures were determined.

The comfort sedation pain (CSP) score was used at regular time intervals to assess patients' comfort, sedation, and pain before and during surgery. Comfort was reported by patients on a scale from 0-5 (0 being the least and 5 being the most comfortable). Sedation was assessed with the adapted Richmond Agitation and Sedation Scale score (Table 1). Pain was assessed with adapted visual analogue scale score (0 meaning no pain, 5 meaning intolerable pain). At the end of procedures, the surgeon’s and anesthesiologist's satisfaction were recorded on a scale from 1 (not satisfied) to 5 (maximum satisfaction).

**Table 1:** Adjusted Richmond Agitation and Sedation Scale (RASS score)

0	Alert and calm	Spontaneously pays attention to caregiver
1	Drowsy	Not fully alert, but has sustained (more than 10 s) awakening, with eye contact to voice
2	Light sedation	Briefly (less than 10 s) awakens with eye contact to voice
3	Moderate sedation	Any movement (but no eye contact) to voice
4	Deep sedation	No response to voice, but any movement to physical stimulation
5	Unarousable	No response to voice or physical stimulation

### Statistical analysis

**Figure 1:**
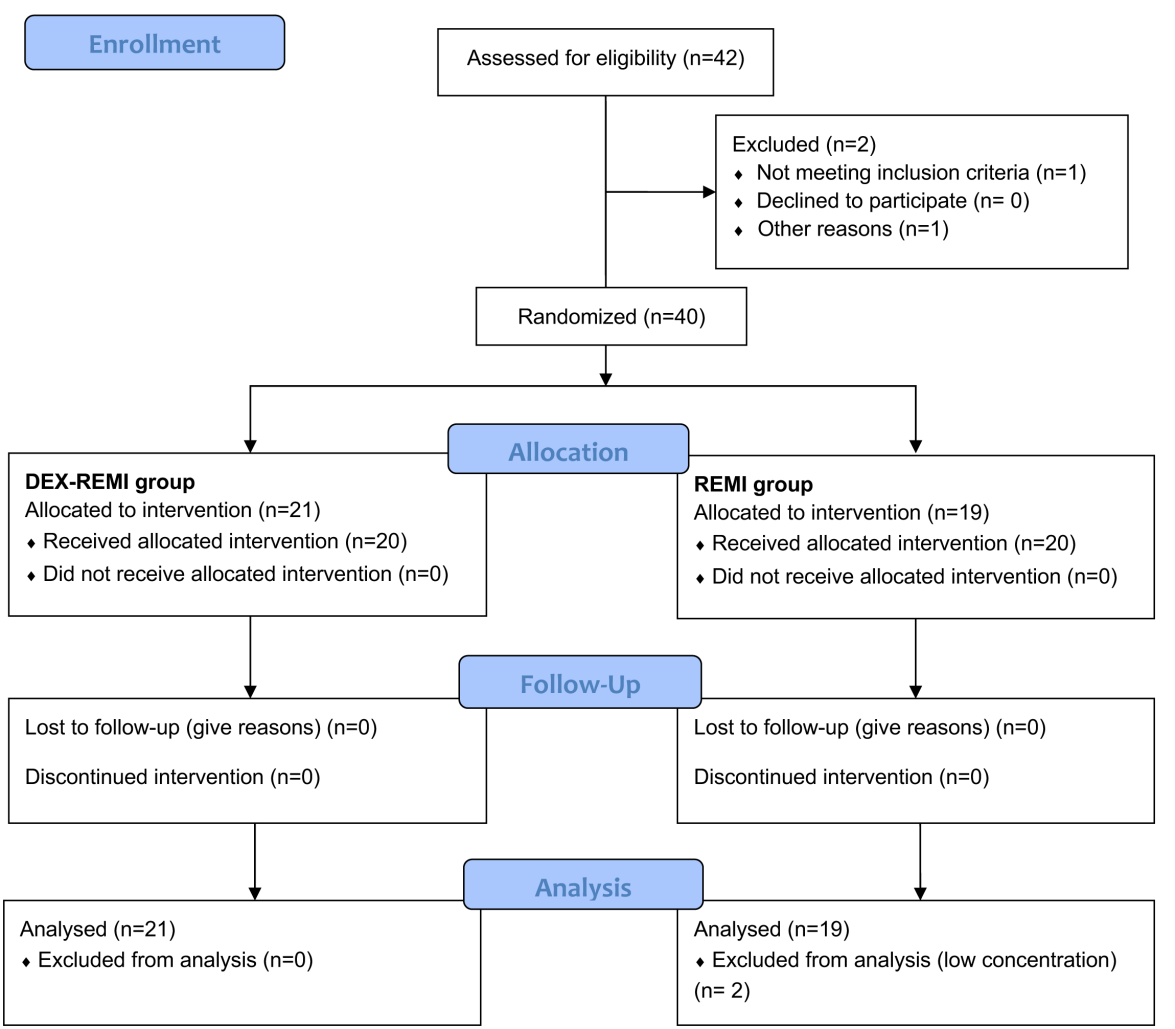
Flow diagram of the study.

The sample size was calculated based on a previous pilot study of two independent groups (5 patients received dexmedetomidine and remifentanil and 5 patients received remifentanil) using a priori two-tailed *t* test power analysis. The difference in the mean remifentanil consumption between the groups was used for the effect size calculation and the resulting minimum sample size determination. For a significance level of 5% (α = 0.05) and a power of 80% (β = 0.2), the calculated minimum sample size was 19. To compensate for possible withdrawals, 21 patients were included in each group. Two patients from the REMI group were excluded from further analysis (Figure 1).

A two-tailed *t* test with unequal variances or the χ^2^ test were used to assess the differences in demographic data, remifentanil and dexmedetomidine doses, hemodynamic parameters, surgical procedure duration, surgeon's, anesthesiologist's, and patients' satisfaction (using the CSP score) and eventual complications. Continuous variables are presented as means, and categorical data are summarized as counts. A *P* value of less than 0.05 was considered significant. Data were analyzed with SPSS 13.0 software (IBM Corp., Armonk, NY, USA).

## RESULTS

The study included 40 patients, 21 sedated with a combination of dexmedetomidine and remifentanil (the DEX-REMI group) and 19 with remifentanil only (the REMI group). A total of 37 patients underwent vitrectomy, 2 underwent silicone band removal, and 1 underwent silicone band implantation.

**Figure 2:**
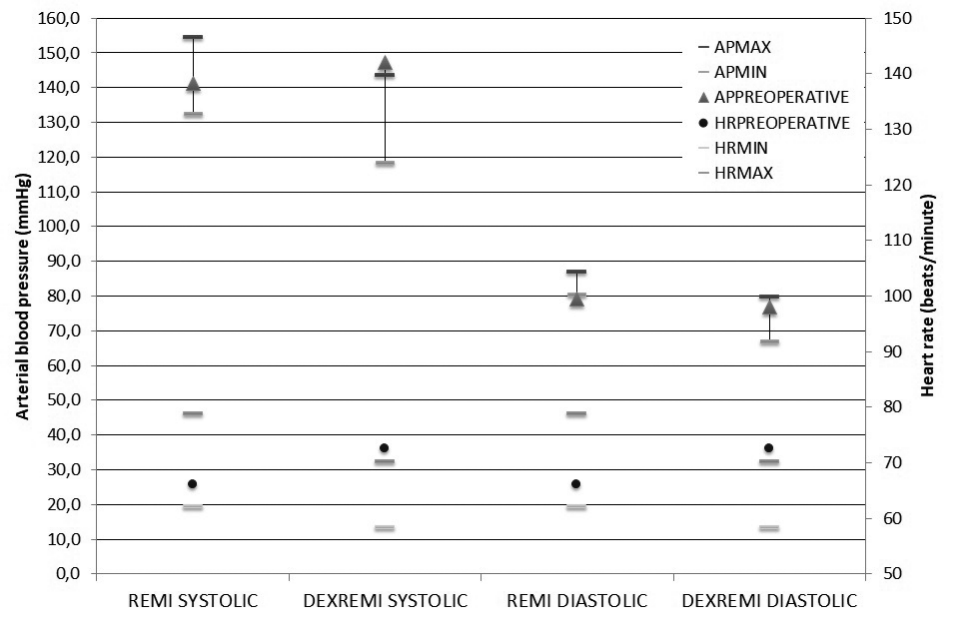
Arterial pressure (AP in mmHg) and heart rate (HR in beats per minute) before the surgery and their minimum and maximum values during the surgery. *P < 0.05: In the dexomedetomidine (DEX)-remifentanil (REMI) group, the mean minimal systolic and diastolic APs were significantly lower than those in the REM group (*P* = 0.04 for systolic and *P* < 0.001 for diastolic).

No significant differences were found between the groups regarding their demographics and duration of surgery (Table 2). Remifentanil consumption was significantly lower in the DEX-REMI group (136.1 vs 288.7 μg; *P* = 0.001). The DEX-REMI group also had significantly lower minimum arterial pressure during surgery (118.3 vs 132.4 mm Hg; *P* = 0.04 for systolic and 76.8 vs 79.1 mm Hg; *P* = 0.00 for diastolic arterial pressure) (Figure 2) and significantly higher satisfaction scores of the surgeon (*P* = 0.01), anesthesiologist (*P* < 0.001), and patients (*P* = 0.05 for comfort and *P* = 0.045 for pain) during the surgery (Table 3). None of the patients had anesthetic complications. The minimum BIS levels ranged from 66% to 90%, with no significant differences between the groups. In the REMI group, 7 patients had nystagmus and 1 suffered an anxiety attack.

**Table 2:** Baseline demographics, drug dosing, and duration of surgical procedure*

	Remifentanil group	Dexmedetomidine group	*P*
Number	19	21	
Age (years)	64 ± 14.1	72 ± 10.2	0.06
Sex (male: female)	12:7	13:8	0.59
Weight (kg)	78.8 ± 19.8	79.8 ± 16.7	0.87
Height (cm)	169.4 ± 13.4	169.8 ± 7.9	0.89
American Society of Anesthesiology score (1/2/3)	0:14:5	1:11:9	0.25
Remifentanil (μg)	288.7 ± 161.3	136.1 ± 110.3	0.001
Dexmedetomidine (μg)	0	195.2 ± 21.8	0
Duration of surgery (min)	52.9 ± 16.7	50.8 ± 14.3	0.67

*The results are expressed as mean ± standard deviation or number of patients.

**Table 3:** Satisfaction of the surgeon and anesthesiologist and comfort sedation pain (CSP) scoring

	Remifentanil group			Dexmedetomidine group			*P*
Surgeon's satisfaction (n: 0-5)	0:0:7:8:4			0:0:3:2:16			0.010
Anesthesiologist's satisfaction (n: 0-5)	0:0:8:9:2			0:0:2:3:16			<0.001
CSP	C	S	P	C	S	P	
Before sedation (CS:0-/5; P: 2-5/0-1)	0/19	19/0	19/0	3/18	21/0	21/0	C:0.200 S:0.500 P:1
After sedation (CS:0-4/5; P: 2-5/0-1)	1/18	18/1	19/0	2/19	21/0	21/0	c: 0.500 s: 0.500 p: 0.700
During the surgery (CS:0-4/5; P: 2-5/0-1)	12/7	18/1	7/12	4/17*	21/0	2/19*	c: 0.005 s: 0.500 p: 0.045
After the surgery (CS:0-4/5; P: 2-5/0-1)	3/16	19/0	1/18	1/20	21/0	0/21	c: 0.400 s: 0.200 p: 0.200

*The results are expressed as mean ± standard deviation or number of patients.

## DISCUSSION

Our study showed that dexmedetomidine was a good alternative for sedation in vitreoretinal surgery. The remifentanil consumption in the DEX-REMI group was significantly lower than that in the REMI group. This is why the rate of side effects and complications connected with remifentanil was lower, and the satisfaction of the patients and surgeon was higher in the DEX-REMI group.

The vitreoretinal procedure is a standard ophthalmic surgery performed under sedation with remifentanil, which requires maximum cooperation from the patient. While the surgeon manipulates the globe, the patient needs to fixate on a target and be cooperative. This fact made us aware of the potential hazard of some neuropharmacological agents such as remifentanil, the regulation of which is a demanding process during the procedure (14). We decided to use dexmedetomidine along with remifentanil to prevent the possibility of an insufficient analgesic effect from dexmedetomidine. The study was double-blind, and the researcher in the operating theater did not know whether a patient received dexmedetomidine or placebo, as the rate of remifentanil infusion was guided by the BIS and patient comfort and pain.

In the present study, we evaluated the level of remifentanil consumption and analgesia provided as the primary outcome. The cumulative dose of remifentanil used along with dexmedetomidine was very low in each patient. This is probably why a lower complication rate was observed in the DEX-REMI group. Additionally, hemodynamic and respiratory effects and higher surgeon's and patients' satisfaction were also observed under dexmedetomidine sedation compared with standard-of-care sedation with a remifentanil infusion only. We noticed that the addition of i.n. dexmedetomidine lowered the minimal systolic and diastolic arterial pressures during surgery. Additionally, we found that none of the patients in the DEX-REMI group had any complications. Thus, we believe that due to its characteristics, i.n. dexmedetomidine brings more satisfaction for patients during shorter surgical procedures than remifentanil alone.

Dexmedetomidine has a more gradual and slower onset of action with i.n. administration than with an i.v. bolus. This may help in avoiding the α1 agonist effects related to higher peak plasma concentrations observed in i.v. administration. Concurrently, the depth of sedation, once it occurs, is comparable between the two methods of administration (15).

Dexmedetomidine has been registered in the USA since 1999 (Precedex; Hospira, Lake Forrest, IL, USA). First, its use was just intravenous and limited to adult intensive care unit (ICU) patients requiring sedation and mechanical ventilation for up to 24 hours. In 2008, dexmedetomidine was additionally allowed in the USA for sedation of non-intubated patients in the perioperative period. Since 2011, dexmedetomidine has been approved in the European Union for the sedation of adult ICU patients requiring a sedation level at which patients remain rousable in response to verbal stimulation (Dexdor; Orion Corporation, Espoo, Finland). Nevertheless, dexmedetomidine has been reported to be useful in many other fields, such as pediatric sedation and as an adjuvant to regional anesthesia methods, supporting its off-label use (16).

The i.n. route is the most used extravascular route of administration for dexmedetomidine in clinical practice (off-label use). Intravenous boluses of dexmedetomidine are not recommended because of possible hemodynamic side effects (hypertension and tachycardia, followed by bradycardia and hypotension). Its labeled use is i.v. infusion, but the pharmacokinetics of this is inappropriate for short procedures such as vitreoretinal surgery, because the steady state and peak concentrations of the infusion are achieved very slowly. The i.v. use of dexmedetomidine in our patients was not appropriate as vitreoretinal procedures are short (the mean duration was 51-53 minutes), and our postoperative care unit was only available for an hour after the operation. According to some case reports and studies performed mainly in the pediatric population, i.n. administration with a special nasal applicator is very useful, and thus, we opted for this route of administration of dexmedetomidine.

Intranasal doses of 1-4 μg/kg dexmedetomidine cause significant sedation, with an onset time of 15-45 minutes, and their effect was observed for 1-2 hours and was well tolerated (17,18). The onset times of i.v. 1 μg/kg dexmedetomidine and i.n. 1 μg/kg dexmedetomidine were 15-20 and 30-45 minutes, respectively (19). Similarly, the depth of sedation, once it occurs, is comparable between the two methods of administration (15,20). The pharmacokinetics and pharmacodynamics of dexmedetomidine are quite unknown, so further research regarding efficacy, safety, and tolerability as well as the optimal administration route, timing, and dosing regimen is required.

Patients in the DEX-REMI group received dexmedetomidine 1 μg per kg of body mass through the i.n. route 30 minutes before surgery. Therefore, the peak plasma concentration of dexmedetomidine was achieved within the first 20 minutes of surgery (13).

The adverse effects of dexmedetomidine include hypotension, hypertension, nausea, and bradycardia, but no such effects have been observed with i.n. administration (21). The minimum arterial pressure during surgery was significantly lower in the DEX-REMI group, but those patients did not need any vasoactive drugs or support. Lower minimum arterial pressure is consistent with the sedative and analgesic properties of dexmedetomidine. Dexmedetomidine is also known for its safe respiratory profile.

The patients who received dexmedetomidine were also more hemodynamically stable than those who received remifentanil only. Because of respiratory depression in the remifentanil group, we had to interrupt the procedure to secure the airways of one patient.

Bradycardia, one of the main dexmedetomidine side effects, was not found in any of our patients, and none of the patients needed atropine.

On the other hand, remifentanil causes respiratory depression, peripheral saturation decrease, bradycardia, and hypotension (22). It has a dose-dependent depressor effect on the respiratory drive. We noted nystagmus in 7 patients receiving remifentanil only; nystagmus is a very rare adverse effect of remifentanil in high doses. It can be pharmacologically induced and can be a hazard to vitreoretinal procedures, as shown in previous case reports, though patients there were treated for depression (23).

In conclusion, sedation with a combination of i.n. dexmedetomidine and an i.v. infusion of remifentanil provides lower remifentanil consumption, better satisfaction scores, and a lower complication rate than sedation with a remifentanil infusion alone. Therefore, i.n. dexmedetomidine is effective and safe in providing adequate procedural sedation for vitreoretinal procedures.
